# Interaction of mTOR and iNOS pathways in protection against Ischemia/Reperfusion injury

**DOI:** 10.22037/ijpr.2019.1100680

**Published:** 2019

**Authors:** Maedeh Arabian, Nahid Aboutaleb, Marjan Ajami, Rouhollah Habibey

**Affiliations:** a *Rajaie Cardiovascular, Medical, and Research Centre, Iran University of Medical Sciences, Tehran, Iran *; b *Physiology Research Center, Physiology Department, Faculty of Medicine, Iran University of Medical Sciences, Tehran, Iran. *; c *Nutrition and Food Technology Research Institute, Faculty of Nutrition Science and Food Technology, Shahid Beheshti University of Medical Sciences, Tehran, Iran. *; d *Department of Neuroscience and Brain Technologies-Istituto Italiano di Technologia,Via Morego, 30, 16163 Genova, Italy.*

**Keywords:** Ischemia/reperfusion, Morphine, iNOS, Oxidative stress, Malondialdehyde, Rapamycin, mTOR

## Abstract

Chronic morphine (CM) treatment increases the phosphorylation of the mammalian target of rapamycin (mTOR), which confers neuroprotection against ischemia/reperfusion (I/R) injury. Besides its important regulatory role in the proliferation, metabolism, and survival of cells, the mTOR is critically involved in intracellular signaling events during I/R injury. In the present study, we investigated the interaction between the expressions of the mTOR and inducible nitric oxide synthase (iNOS) and their possible protective effects on hippocampal neurons against I/R injury in morphine-dependent mice. Additive doses of morphine were administered for 5 days to* BALB/c *mice so as to induce CM preconditioning before I/R injury. Global brain ischemia was induced via the occlusion of bilateral common carotid arteries for 30 min. CM attenuated iNOS expression, NO production, and malondialdehyde activity in the hippocampal tissue. Pretreatment with rapamycin, the inhibitor of mTOR, abolished all the above mentioned effects of CM. These findings suggested that CM acted through the mTOR signaling pathways to regulate iNOS expression and oxidative state in the hippocampal tissue after I/R injury.

## Introduction

Ischemia/reperfusion (I/R) injury to the tissue can be augmented by preceding episodes of brief and sublethal ischemia, termed “ischemic preconditioning” (1). Ischemic preconditioning triggers different signaling pathways to increase tissue tolerance against severe ischemic insult. These molecular pathways can be triggered by pharmacological agents such as volatile anesthetics , and morphine (2). Several studies on different tissues have confirmed the protective effects of morphine and other opioid-receptor agonists (3-5). In the heart and kidney, it has been shown that treatment with chronic morphine (CM) confers more considerable protective effects than acute or single doses of morphine (4, 6, 7). Further, there is an evidence that chronic and acute morphine treatments may act through different signaling pathways to boost tissue tolerance against I/R injury (8). Several molecular pathways have been suggested for morphine preconditioning including upregulation of heat shock proteins and Bcl-2, mitogen-activated protein kinases (9, 10), mitochondrial ATP-sensitive potassium (KATP) channels (11, 12), and mammalian target of rapamycin (mTOR) (13). 

The mTOR is an evolutional serine/threonine kinase involved in the migration, protein synthesis, proliferation, and autophagy of cells (14). It has been shown that the mTOR has a crucial role in preconditioning against I/R injury (13). Conversely, some studies have indicated that the inhibition of the mTOR signaling pathway by rapamycin (Rapa) decreases neural damage in oxygen- and glucose-deprived conditions (15, 16). A previous investigation demonstrated that in the cardiac tissue, Rapa pretreatment produced cardioprotective effects by opening mitochondrial KATP channels (17). Another study showed that Rapa decreased the expression of inflammatory factors, including inducible nitric oxide synthase (iNOS) and tumor necrosis factor-alpha, in oxygenglucose deprivation/reoxygenation astrocytes (18). However, the mTOR is one of the upstream targets of ribosomal protein S6 kinase beta-1 (p70S6k) and downstream targets for protein kinase B (Akt), which are among the well-studied signaling pathways against I/R injury. Therefore, the mTOR as a regulatory protein for Akt can also be considered an important player in neuroprotection against I/R injury (19). 

Previous evidence suggests a link between p70S6k activation, NO production, and iNOS expression in oxidative stress (20, 21). Generally, the regulation of iNOS expression is at transcriptional level through the activation of some transcription factors such as interferon regulatory factor 1, nuclear factor-kappa beta, and signal transducer and activator of transcription 1 (22). These transcription factors induce iNOS protein expression and NO production via binding to the promoter of the iNOS gene (23).

Regardless of the controversies on the negative or positive effects of the mTOR on I/R injury (24, 25), its role and involvement in the process of oxidative stress and reactive oxygen species (ROS) production after cerebral ischemia or ischemic/pharmacological preconditioning is still unknown. A possible solution to such controversies is to understand the interaction between the mTOR and well-studied signaling pathways involved in tissue injury or tolerance, including mitochondrial KATP channels and NO signaling pathways. Following up on our previous findings regarding morphine-induced tissue tolerance through the mTOR phosphorylation, we designed the present study to assess the interaction between the mTOR and NO production and iNOS expression. 

*Method*

Adult male *BALB/c *mice (weight = 25–30 g) were purchased from Pasteur Institute (Tehran, Iran) and housed in an animal facility with standard conditions of a 12-h light/12-h dark cycle and free access to chow and water. All the experimental procedures were approved by the Ethics Committee of Iran University of Medical Sciences in accordance with the guidelines of the National Institutes of Health for the care and use of laboratory animals (NIH Publications No. 8023, revised 1985).

The animals were divided into 6 groups, 12 mice per group, as follows:

- Sham: Injection of normal saline for 5 days, followed by surgery without bilateral common carotid artery occlusion.
- CM+Sham: Administration of morphine for 5 days, followed by sham surgery.
- I/R: Injection of normal saline for 5 days, followed by 30 minof ischemia.
- CM+I/R: Administration of morphine for 5 days, followed by 30 min of ischemia.
- Rapa+I/R: Injection of normal saline for 5 days, followed by 30 min of ischemia along with the administration of Rapa (5 mg/kg) before the surgical procedure.
- CM+Rapa+I/R: Administration of morphine for 5 days, followed by 30 minutes of ischemia along with the administration of Rapa (5 mg/kg) 30 min after the last morphine dose.

*Chronic Morphine Treatment*

For the induction of morphine preconditioning, cumulative doses of morphine were administrated subcutaneously for 5 consecutive days. The preconditioning regimen was 10 mg/kg/d on the first and second days and 15 mg/kg/d on the third and fourth days. All the daily doses were injected twice a day at 9:00 AM and 5:00 PM. On day 5, a final dose of 30 mg/kg was given 4 hbefore ischemia. This method for the induction of morphine preconditioning has already been validated in CM-treated rat and mouse models (7, 26). The control groups received a normal saline solution instead of morphine. Rapa (5 mg/kg) was also administered 30 minutes after the last dose of morphine and 3.5 h before I/R induction. 

*Ischemia/Reperfusion Procedures*

The mice were deeply anesthetized with a mixture of xylazine (10 mg/kg) and ketamine (50 mg/kg) 4 h after the last dose of morphine. Body temperature was monitored with a rectal probe and maintained at 36 ± 0.5 °C by using a heating pad. The right and left common carotid arteries were exposed through a neck incision and dissected from the vagus nerve and the surrounding tissues. Bilateral occlusion of the common carotid arteries was performed using a microsurgery clamp for 30 min. After the release of the clamp, a 24-h period of reperfusion was allowed to collect the hippocampal tissue for the evaluation of enzymatic activity and protein expression (26).

**Figure 1:**
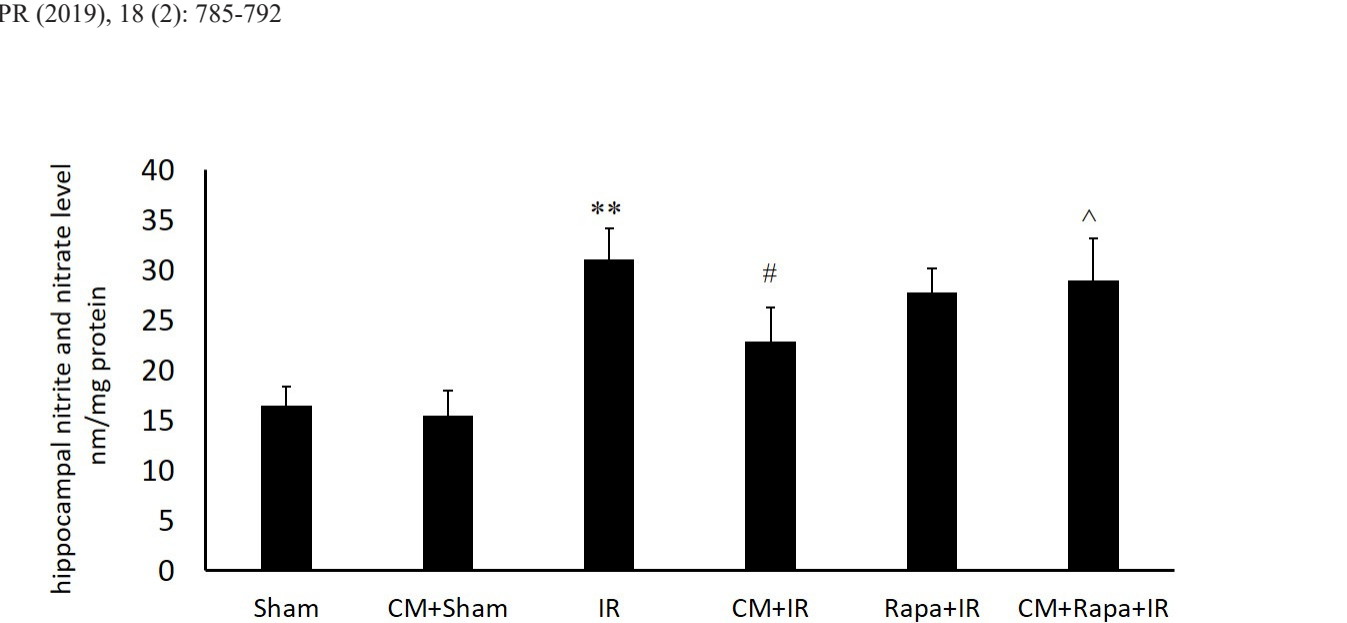
Tissue levels of nitric oxide metabolites in the different groups

**Figure 2:**
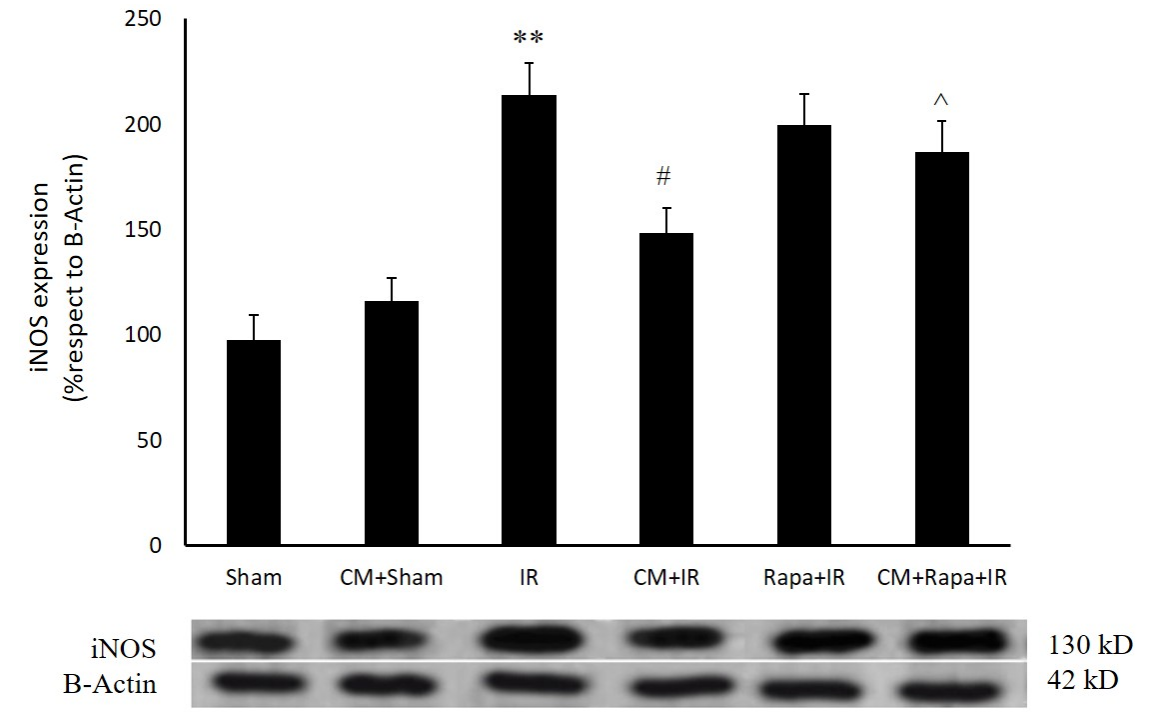
Inducible nitric oxide synthase (iNOS) protein expression in the different groups

Percentages of the iNOS/β-actin expression (ratio of the expression of each protein to the expression of β-actin in the same sample calculated based on the optical density) are presented. Data are shown as mean ± SEM. ** P < 0.01 vs the sham group, # P < 0.05 vs the I/R group, and ^ P < 0.05 vs the CM+I/R group.

**Figure 3:**
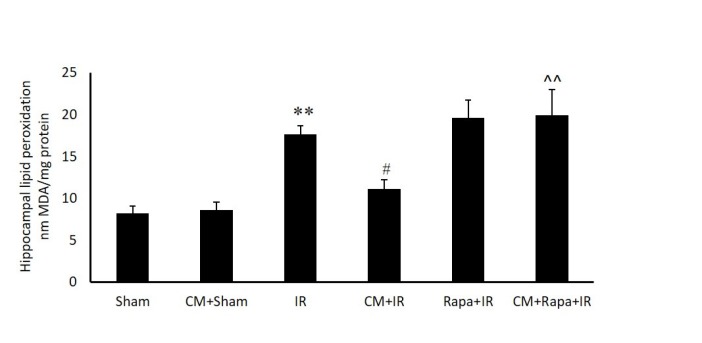
Tissue levels of malondialdehyde (MDA) in the different groups

Data are shown as mean ± SEM. ** P < 0.01 vs the sham group, # P < 0.05 vs the I/R group, and ^^ P < 0.01 vs the CM+I/R group.

*Western Blot Analysis*

The expression of the iNOS protein was measured by deeply anesthetizing the mice 24 h after I/R. The hippocampal tissue was immediately dissected after the removal of the brain and stored in sealed vials at – 70 °C. On the day of the experiment, the hippocampal tissue was lysed on ice in a RIPA lysis buffer for 30 min and centrifuged at 13000*g* (4 °C for 20 min). A spectrophotometer was used to measure the protein concentrations. After the samples were boiled with a loading buffer at 95 °C for 5 min, the proteins were separated on polyacrylamide sodium dodecyl sulfate gels (10%) and then transferred to nitrocellulose membranes. The membranes were blocked with 5% milk in Tris-buffered saline for 1 h and incubated at 4 °C overnight with primary antibodies (Cell Signaling Technology, Italy). The primary antibodies were the rabbit polyclonal antibody for iNOS (#2982, Rabbit polyclonal antibody for iNOS, Cell Signaling Technology, Italy) and β-actin as the loading control (Abcam; ab8226). Thereafter, the membranes were washed and incubated with the horseradish peroxidase-conjugated anti-mouse secondary antibody (Cell Signaling Technology; #7072) for 1 h at room temperature. Finally, the protein bands were scanned and the expression level of each protein was normalized to the β-actin expression and analyzed with Lab Work software.

*Determining Nitric Oxide Metabolite Concentrations in the Hippocampal Tissue*

Tissue NO levels (total nitrite) were quantified via the Griess reaction after the incubation of the supernatant with nitrate reductase to convert NO_3_ to NO_2_. The Griess reagent (0.1% naphthylethylenediamine hydrochloride, 1 mL of 1% sulfanilamide, and 2.5% phosphoric acid) was then added to 1 mL of the supernatant. After incubation for 30 min, absorbance was read at 545 nm (UV mini-1240 spectrophotometer, Shimadzu Corp, Kyoto, Japan) and the total nitrite concentration was subsequently calculated by comparing the absorption intensity with the standard curve (27, 28).

*Malondialdehyde Activity Assessment Protocol*

Twenty-four h after brain ischemia, the hippocampal samples were homogenized in a RIPA buffer containing a protease inhibitor. After centrifuging, the supernatant was removed and used to determine malondialdehyde (MDA) levels. MDA was measured based on the thiobarbituric acid method via the MDA Assay (Northwest NWK-MDA01) (29). The red light of the mixture was read at a wavelength of 532 nm. The obtained results were calculated from the standard curve (2.5, 5, 10, and 20 nMol/mg) and expressed as nMol/mg protein.

*Statistical Analysis*

SPSS statistical software, version 16.0 (IBM Co, USA), was used for the statistical analyses of the obtained data. All the data were analyzed using one-way ANOVA, followed by the post-hoc Tukey test for further analysis on between-group comparisons. The results were expressed as mean ± standard error of the mean, and a *P *value less than 0.05 was considered statistically significant.

## Results

*Tissue Levels of Nitric Oxide*

The NO metabolites were increased post I/R (31.13 ± 3.15 vs the sham group with 16.51 ± 1.86; *P *< 0.01) (Figure. 1), implying the extensive production of NO in the hippocampal tissue following global brain ischemia. Conversely, in the CM-treated mice, the NO level in the hippocampal tissue was decreased subsequent to ischemia (15.5 ± 2.44 vs the I/R group; *P *< 0.05) (Figure. 1), which was reversed with Rapa pretreatment (28.9 ± 4.2 vs the CM+I/R group; *P *< 0.05) (Figure. 1).

*Effects of Chronic Morphine on the Expression of the Inducible Nitric Oxide Synthase Protein*

As is shown in Figure 2, twenty-four h post ischemia, the expression of the iNOS protein exhibited a significant rise (213 ± 15.24%; *P *< 0.01 vs 97.7 ± 11.5% in the sham group). CM had no effect on iNOS expression in the sham-operated group; however, it decreased iNOS expression 24 h post ischemia (148.4 ± 11.91% vs the I/R group; *P *< 0.05) (Figure. 2). Rapa treatment before ischemia blocked the effect of CM on iNOS expression (186.9 ± 14%; *P *< 0.05 vs the CM+I/R group) (Figure. 2).

*Malondialdehyde Activity*

The possible involvement of oxidative stress in the hippocampal tissue was confirmed by measuring the MDA level in the different groups (expressed as nMol/mg tissue protein). I/R significantly increased MDA in the hippocampal tissue in comparison with the sham group (19.54 ± 0.96 vs 9.33 ± 0.73 in the sham group; *P *< 0.01) (Figure. 3). CM alone did not change the MDA level, whereas CM significantly decreased the amount of MDA in the I/R group (13.13 ± 1.11; *P *< 0.05). The administration of Rapa prior to I/R in the CM-treated mice prevented the effects of CM on decreasing MDA (19.95 ± 3.02 vs the CM+I/R group; *P *< 0.01). Rapa treatment did not change the MDA level in the I/R group (Figure. 3).

## Discussion

The results of the present study in the first step confirmed the modulatory effect of CM treatment on the iNOS-NO axis, reflected as neural protection after I/R injury. The pretreatment of the mice with Rapa significantly inhibited the protective effects of CM, which was concomitant with increased iNOS expression, NO production, and MDA level in the hippocampal tissue. These results suggested an interaction between the mTOR pathway, oxidative stress, and iNOS-NO signaling axis, all of which are also actively involved in CM-induced neuroprotection. 

Understanding the exact cellular mechanisms responsible for iNOS activation can reveal novel therapeutic targets for interventions in NO-induced inflammatory diseases. Previous research shows that iNOS expression is regulated by complex mechanisms at molecular level, including transcriptional and post-transcriptional processes (30). An important post-transcriptional regulatory mechanism is the modulation of the stability of iNOS mRNA by numerous RNA-binding proteins (31). Interestingly, the mTOR kinase regulates the function of a few RNA-binding proteins (32). In our previous study, we succeeded in demonstrating that Rapa abolished the neuroprotective effects of CM preconditioning and lessened superoxide dismutase enzyme activity, which led to apoptosis in hippocampal neurons (13). It has been shown that Rapa and its analog, RAD001, attenuate iNOS expression in microglial activation by pro-inflammatory cytokines, while they exert no effects on iNOS expression in astrocytes (33). Such evidence suggests that the mTOR may act as a double-edged sword on regulating oxidative stress and inflammatory response in the nervous system. Previous studies have suggested both inhibitory and stimulatory effects of the mTOR on iNOS expression (34). 

p70S6k is a serine/threonine kinase, the mTOR downstream target, which is regulated by the PI3k/mTOR pathway. The PI3k/Akt/mTOR signaling pathway has a potential interaction with the iNOS/NO pathway (35). Recently, several studies have demonstrated the important role of the p70S6k and PI3k pathways involving Akt in NO production (36). Moreover, it has been revealed that the active mTOR is required for the phosphorylation of p70S6k and 4E-binding protein-1 and that these phosphorylations can be abolished by Rapa and result in an imbalance in NO production and iNOS expression (37). Our results in the present study showed that Rapa by the inhibition of the mTOR induced an excessive increase in iNOS expression and consequently augmented NO production following I/R injury and abolished the CM-induced neuroprotection. Therefore, the mTOR pathway seems crucial for the balance between NO production and iNOS expression following I/R injury in CM-treated mice, which implies an anti-oxidative role for the mTOR. Our results are in contrast with the previous studies performed on other cell types such as macrophage, astrocytes (34), and myoblasts (38), which showed that the mTOR inhibition decreases iNOS expression and NO production. Notwithstanding the active interplay between the mTOR pathway and the iNOS-NO axis, it seems that these interactions are mainly dependent on the activating stimulus or the cell type. In our previous investigation, we showed that morphine-induced neuroprotection at hippocampal neurons was accomplished by the mTOR phosphorylation, which consequently decreased apoptosis and its effects were reversed in the presence of Rapa (13). The mTOR phosphorylation at Ser2448 is a key marker of the mTOR pathway activation (33). Usually under ischemic conditions, the tissue NO level rises owing to iNOS upregulation. Therefore, nitrite levels could be an indirect indicator of iNOS activity. In tandem with our assessment of NO levels, we also evaluated the hippocampus level of the MDA enzyme. Excessive ROS production after I/R injury is the main factor responsible for cellular apoptosis and loss. ROS act as signaling mediators that are involved in mitochondria and DNA repair enzymes and lead to apoptosis (39, 40). The salient point is that the level of ROS released during I/R injury is much more than the endogenous antioxidant capacity (41-43). Tissue levels of MDA, as an index of oxidative tissue damage and ROS production, clearly indicate the outcome of CM treatment and the mTOR inhibition after I/R injury.

## Conclusion

Our findings strongly suggested that CM downregulated I/R-induced iNOS expression and modified oxidative stress and NO production through the activation of the mTOR signaling pathway. Rapa, an mTOR inhibitor, augmented iNOS expression following I/R injury and abolished the effects of CM on neuronal survival, implying an interplay between the mTOR pathway and the iNOS-NO axis in morphine-induced neuroprotection.
